# Foot orthoses and physiotherapy in the treatment of patellofemoral pain syndrome: A randomised clinical trial

**DOI:** 10.1186/1471-2474-9-27

**Published:** 2008-02-27

**Authors:** Bill Vicenzino, Natalie Collins, Kay Crossley, Elaine Beller, Ross Darnell, Thomas McPoil

**Affiliations:** 1 Division of Physiotherapy, School of Health and Rehabilitation Sciences, University of Queensland, Brisbane, Australia; 2 School of Physiotherapy, The University of Melbourne, Melbourne, Australia; 3 Queensland Clinical Trials Centre, School of Population Health, University of Queensland, Brisbane, Australia; 4 Gait Research Laboratory, Department of Physical Therapies, Northern Arizona University, Flagstaff, USA

## Abstract

**Background:**

Patellofemoral pain syndrome is a highly prevalent musculoskeletal overuse condition that has a significant impact on participation in daily and physical activities. A recent systematic review highlighted the lack of high quality evidence from randomised controlled trials for the conservative management of patellofemoral pain syndrome. Although foot orthoses are a commonly used intervention for patellofemoral pain syndrome, only two pilot studies with short term follow up have been conducted into their clinical efficacy.

**Methods/design:**

A randomised single-blinded clinical trial will be conducted to investigate the clinical efficacy and cost effectiveness of foot orthoses in the management of patellofemoral pain syndrome. One hundred and seventy-six participants aged 18–40 with anterior or retropatellar knee pain of non-traumatic origin and at least six weeks duration will be recruited from the greater Brisbane area in Queensland, Australia through print, radio and television advertising. Suitable participants will be randomly allocated to receive either foot orthoses, flat insoles, physiotherapy or a combined intervention of foot orthoses and physiotherapy, and will attend six visits with a physiotherapist over a 6 week period. Outcome will be measured at 6, 12 and 52 weeks using primary outcome measures of usual and worst pain visual analogue scale, patient perceived treatment effect, perceived global effect, the Functional Index Questionnaire, and the Anterior Knee Pain Scale. Secondary outcome measures will include the Lower Extremity Functional Scale, McGill Pain Questionnaire, 36-Item Short-Form Health Survey, Hospital Anxiety and Depression Scale, Patient-Specific Functional Scale, Physical Activity Level in the Previous Week, pressure pain threshold and physical measures of step and squat tests. Cost-effectiveness analysis will be based on treatment effectiveness against resource usage recorded in treatment logs and self-reported diaries.

**Discussion:**

The randomised clinical trial will utilise high-quality methodologies in accordance with CONSORT guidelines, in order to contribute to the limited knowledge base regarding the clinical efficacy of foot orthoses in the management of patellofemoral pain syndrome, and provide practitioners with high-quality evidence upon which to base clinical decisions.

**Trial registration:**

Australian Clinical Trials Registry ACTRN012605000463673

ClinicalTrials.gov NCT00118521

## Background

Patellofemoral pain syndrome (PFP) is a distinct clinical entity defined as "idiopathic pain arising from the anterior knee/patellofemoral region that is of otherwise unknown origin" [1]. Falling within the classification of lower limb musculoskeletal overuse conditions, PFP is highly prevalent among active individuals. A recent retrospective review of running injuries found PFP to be the most common presentation to a sports medicine clinic in both females (19.2% of injuries) and males (13.4% of injuries) [2]. The pain and disability resulting from this condition not only affects short term participation in daily and physical activities, but can have a significant long term impact, with symptoms shown to persist in 1 in 4 sufferers for up to 20 years after initial presentation [3]. As regular physical activity is highly recommended for the prevention of conditions such as cardiovascular disease and type II diabetes, PFP may have important implications for the long term health of affected individuals.

Given the prevalence and impact of this condition and the subsequent demands placed on health care practitioners, the lack of high quality research on the conservative management of PFP is surprising [4,5]. A recent systematic review by Crossley et al [5] concluded that current management of PFP is not based on evidence from randomised controlled trials (RCTs), which are widely deemed to be the gold standard of research design in providing the best evidence for health care interventions [6].

Foot orthoses are a commonly used and frequently recommended intervention in the management of PFP [7]. Preliminary evidence of their clinical efficacy is provided by findings of a pilot study of 20 adolescent females [8]. The addition of soft foot orthoses to an exercise program resulted in significantly greater improvements in pain than treatment with flat insoles and exercises over eight weeks. A more recent study by Wiener-Ogilvie & Jones [9] however found no difference in outcome between 8 weeks of treatment with functional foot orthoses, exercises, or orthoses with exercises. The authors considered the small sample size (N = 31) to have contributed to the inability to detect a significant difference between the three groups. Conversely, a popular physiotherapy program used routinely in Australia does have high quality RCT evidence to support its use in PFP. Crossley et al [10] showed this six week physiotherapy program of quadriceps muscle retraining, patellofemoral joint mobilisation, patellar taping and daily home exercises to be significantly superior to sham ultrasound, non-therapeutic gel application and placebo taping. The short follow up period used in all three studies could be considered to be a downfall, particularly given the condition's demonstrated tendency for chronicity [3].

In order to provide clinicians with high quality evidence upon which to base clinical decision making, it is therefore timely to conduct a RCT to establish the long term clinical efficacy of foot orthoses in the management of PFP.

## Methods

### Aim

The primary aim of this study is to determine the clinical efficacy of foot orthoses in the management of PFP, as compared to a flat insole, a proven physiotherapy program, and a combined intervention of foot orthoses and physiotherapy. The cost-effectiveness of foot orthoses compared to the other interventions will also be evaluated.

### Study design

**Figure 1:**
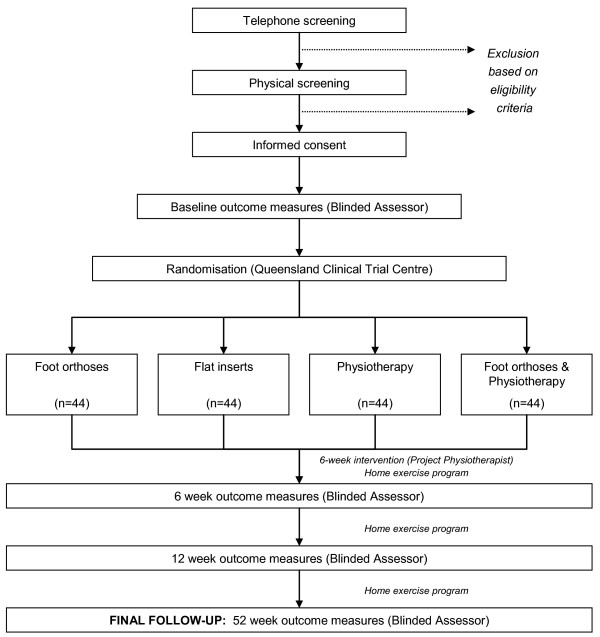
Flow of participants through the randomised clinical trial.

The above aims will be investigated through the conduct of a pragmatic randomised single-blinded clinical trial in a community setting over a 12 month period. All participants will provide written informed consent prior to randomisation. The investigator responsible for taking the outcome measures will be blinded to participants' group allocation. In order to maintain blinding of this investigator, randomisation will be effected and controlled by an independent body, and a research assistant will perform all communication regarding group allocation. An overview of the study protocol is provided in Figure 1.

### Ethics

Ethical approval for this study has been granted by the University of Queensland's Medical Research Ethics Committee.

### Eligibility criteria

Volunteers will be eligible for participation in the study on the basis of the following criteria: (1) anterior or retropatellar knee pain of non-traumatic origin and greater than six weeks duration that is provoked by at least two of the following activities: prolonged sitting or kneeling, squatting, jogging or running, hopping, jumping, or stair ascending/descending; (2) the presence of pain on palpation of the patellar facets, on step down from a 25 cm step, or during a double leg squat; and (3) pain over the previous week equal to or greater than 30 mm on a 100 mm visual analogue scale. Due to the nature of the outcome measures to be used, participants will require an acceptable understanding of written and spoken English. An ability and willingness to attend all sessions required for completion of the study and comply with intervention protocols for the 12 month study period will also be an essential criterion. Volunteers will be excluded if they have any of the following: (1) concomitant injury or pathology of other knee structures (e.g. menisci, collateral and cruciate ligaments, patellar tendon, iliotibial band, pes anserinus); (2) a history of knee surgery, patellofemoral dislocation or sublaxation, Osgood-Schlatter's disease or Sinding-Larsen-Johanssen syndrome; (3) a positive patellar apprehension test; evidence of knee joint effusion; (4) any foot condition that may preclude the use of foot orthoses; (5) pain in and/or referred from the hip or lumbar spine; or (6) a known allergy to rigid strapping tape. Prior treatment with foot orthoses will also preclude volunteers from participating in order to facilitate blinding as to the differences between the foot orthoses and flat insoles, as will prior physiotherapy treatment for PFP within 12 months of entry into the study and current use of anti-inflammatories or corticosteroids. All participants will be required to be a minimum of 18 years of age for consent purposes, and a maximum of 40 years in order to minimise the likelihood of degenerative joint changes.

### Recruitment of study participants

In order to recruit a representative sample of sufficient size, a multifaceted recruitment strategy that has been successful in past clinical trials will be used. This will target the greater Brisbane, Gold Coast and Toowoomba regions in Queensland, Australia. Paid advertisements in local and regional newspapers will be placed at regular intervals during the recruitment period, along with media releases to broaden the scope to radio and television media. These will be reinforced by regular posting of advertisements on University, gymnasium and community noticeboards within the catchment area. It is anticipated that a small number of referrals will also come from physiotherapists involved in the study, as well as general practitioners through the provision of information and advertising packages.

Volunteers who respond to advertisements will be put through a two-stage screening process to determine their suitability for inclusion in the study. Firstly, a preliminary telephone interview will be conducted to screen for major exclusion criteria. Potential participants will then be invited to attend an appointment at the University of Queensland, where they will be provided with further information about the screening process and study. A comprehensive musculoskeletal examination will then be conducted by a qualified physiotherapist to determine the volunteer's suitability for inclusion based on the eligibility criteria. At the completion of the examination, eligible volunteers will be provided with an information sheet that thoroughly explains the study protocol. They will then attend a follow-up appointment for completion of informed consent documentation and baseline outcome measures. This will be conducted by an assessor who will remain blind to group allocation and be responsible for taking outcome measures at designated follow-up times. At this appointment, the knee rated to be the most severe by participants with bilateral PFP will be selected as the knee to be studied.

### Randomisation

Once informed consent has been obtained and baseline outcome measures completed, each participant will be assigned a participant number and randomly allocated to one of four intervention groups via concealed allocation. The Queensland Clinical Trial Centre, an independent off-site body, will be responsible for generating and maintaining the randomisation sequence. A research assistant will perform the communication between the Queensland Clinical Trial Centre, participants and project physiotherapists to prevent unblinding of the blinded assessor.

### Intervention

Interventions will be administered by one of 17 experienced registered physiotherapists (Physiotherapists Board of Queensland) located throughout the catchment area. All project physiotherapists will have attended two seminars for explanation and discussion of the intervention protocols and training in fitting of foot orthoses. A qualified sports physiotherapist who has extensive experience in the management of PFP with orthotics will conduct these seminars. A comprehensive manual outlining study procedures will be provided to all project physiotherapists.

Participants will attend six 20–60 minute appointments over a six week period (i.e. one session per week), with details of each session recorded by the project physiotherapist in a treatment log. On completion of the six visits, participants will continue with a self-management program for the remainder of the 12 month study period. This will incorporate the home exercise program relevant to their allocated intervention.

The four intervention groups are as follows.

#### 1. Foot orthoses

**Figure 2:**
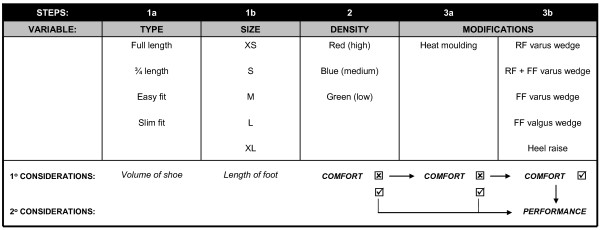
Sequential procedure for fitting of foot orthoses.

Participants assigned to this group will be provided with prefabricated foot orthoses from a commercially available range (Vasyli International). The orthoses are made of ethylene-vinyl acetate (EVA) of high (Shore A 75°), medium (Shore A 60°) or low (Shore A 52°) density, and have an inbuilt arch support and 6° varus wedge as specified by the manufacturer. The range includes four types of orthoses to suit a variety of footwear: full length and three-quarter length (general use); easy fit (a three-quarter length with lateral cut-away for narrower footwear e.g. men's dress shoes); and slim fit (a thin minimally arched orthosis designed for women's dress/court shoes). In order to maximise compliance, orthoses will be fitted to up to four pairs of the participants' shoes, using a standardised iterative procedure based primarily on perceived comfort (Figure 2). The first stage will involve selection of an appropriate device based on the amount of space available in the shoe (Step 1a) and the length of the participant's foot (Step 1b). In the first instance, an orthosis made from the highest density EVA will be used (Step 2). Following this process, if the orthoses are uncomfortable when worn in the shoe, the therapist will review the type, size and density, and then choose an alternative orthosis that does fit comfortably. If the participant is not satisfied with the comfort level of the orthoses, the orthoses will be customised. This will first involve heat moulding as per the manufacturer's instructions (Step 3a). This process involves mildly heating the underside of the orthosis, placing it in the intended shoe and then having the participant stand on it with the foot positioned in its neutral zone for approximately 60 seconds. If the heat moulding process alone fails to optimise comfort, the therapist will sequentially trial the addition of medial wedges of the rear and forefoot and/or a heel raise to the orthoses (Step 3b). The medial rearfoot wedges have a manufacturer specified 2° or 4° of inclination, the forefoot wedges 4° or 6° and the heel raises are 4, 6 or 8 mm thick. The forgoing underscores the primary goal of fitting of the orthoses to achieve a comfortable fit. Once comfort has been achieved, the effect of the orthoses on performance of a functional task will be evaluated. This will involve quantification of the effect of the orthoses on pain-free performance of an activity identified as pain provocative immediately prior to orthosis fitting, such as, step ups, step downs or squats. A substantial increase in the number of pain-free repetitions that can be performed will be regarded as a success. The therapist will modify the orthoses in order to improve the performance of the functional task, but with the fundamental aim of ensuring the orthoses are comfortable. This process of fitting, reviewing and adjusting the orthoses will continue over the six visits. Participants will also be given a home exercise program of foot arch-forming exercises and weight-bearing calf stretches to be performed bilaterally twice daily.

#### 2. Flat inserts

This condition will act as a control for the application of inserts into the footwear, and will likely account for some of the potential placebo effects of prescribing an in-shoe device. The flat inserts will be identical in initial appearance to the foot orthoses described above, made from the same EVA with identical covering fabric and company logo. However, this device will be of uniform thickness along its length (3 mm) and have no inbuilt arch or varus wedging. The only modification that will be made to this insert is gentle heat moulding over the six appointments. Participants will be taught a home exercise program of minimal balance training, slowly progressing from standing on one leg while holding a rail for support, to unsupported single leg stance, moving toes up and down, and with eyes closed. The exercise program will not be reinforced as with the other interventions.

#### 3. Physiotherapy

**Figure 3:**
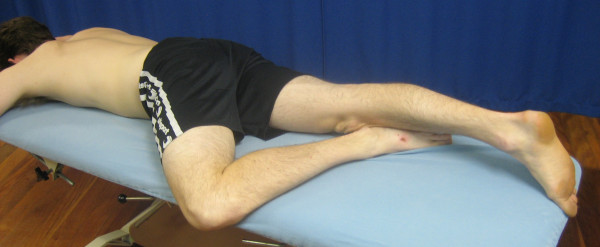
'Figure 4' anterior hip stretch in prone.

**Figure 4:**
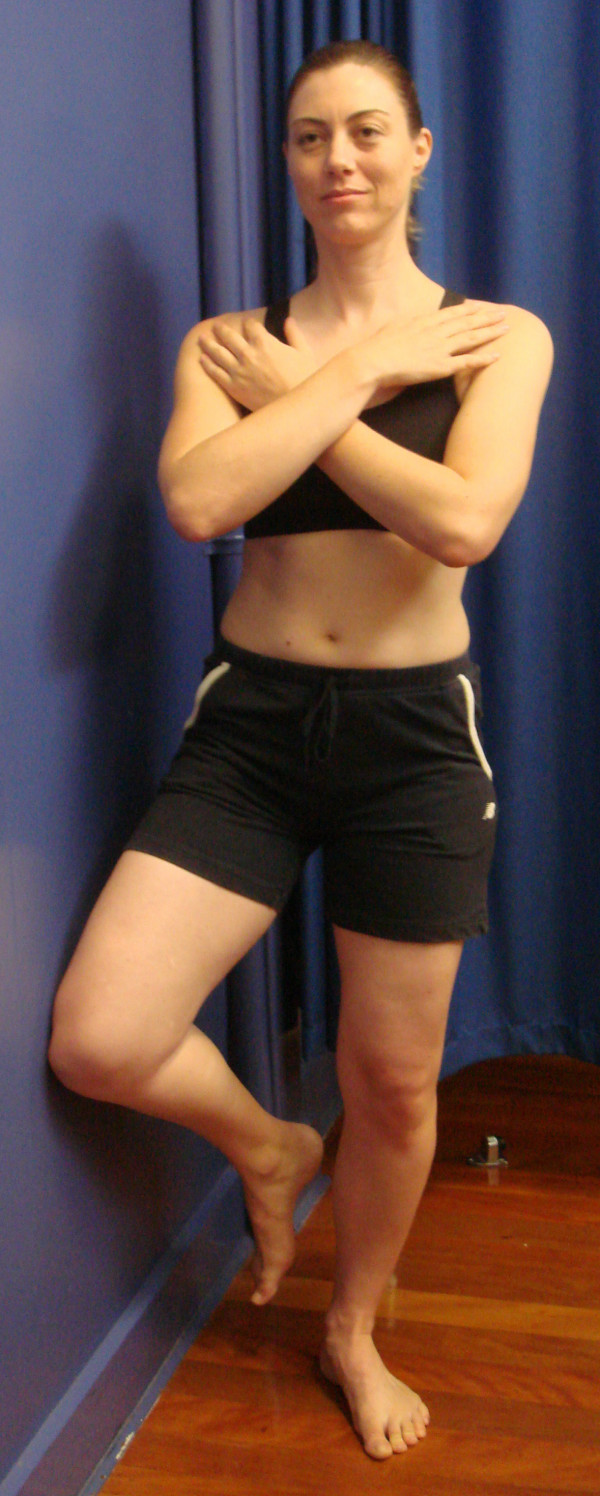
Hip external rotation retraining.

The physiotherapy program will consist of a combined therapy approach that represents the current Australian best practice. A recent RCT showed this program to produce a significantly greater reduction in pain and disability over six weeks compared to a placebo intervention [10]. The major components are outlined in Table 1 and illustrated in Figures 3 and 4.

**Table 1:** Physiotherapy program (adapted from Crossley et al, 2002).

**Intervention**	**Dose**
***Patellar mobilisation:***	
I. Passive patellar medial glide and tilt combined with transverse friction massage of the lateral retinaculum	4 × 30 seconds
	
***Stretches:***	
I. Hamstring stretches	3 × 20 seconds; bilateral
II. 'Figure 4' anterior hip stretch (Figure 3)	3 × 20 seconds; bilateral
	
***Patellar taping:***	
I. Taping of the patella:	Daily application for 6 weeks
1. Medial tilt and posterior tilt	
2. Medial glide and posterior tilt	
3. Fat pad unloading	
4. Medial rotation	
	
***Exercises:***	
I. Hip external rotation retraining (Figure 4)	3 × 20 seconds; bilateral
II. Vasti retraining (with EMG biofeedback):	
i. Isometric VMO contraction in sitting	3 × 10 repetitions (affected);
	1 × 10 repetitions (unaffected)
ii. Inner range knee flexion in standing	3 × 10 repetitions
iii. Step downs (when able to perform 5 pain-free repetitions):	
a) Slow eccentric lowering on affected leg from 10 cm step	3 × 10 repetitions
b) Increased step height (20 cm step)	3 × 10 repetitions
c) Alternating speed (down slow, up fast; down fast, up slow)	3 × 10 repetitions
	
***Home exercise program:***	
I. Self-mobilisation of the affected patella	As above; twice daily
II. Patellar taping (as above)	
III. Stretches (as above)	
IV. Exercises (as above)	

#### 4. Foot orthoses and Physiotherapy

Participants assigned to this group will receive both the foot orthoses and physiotherapy programs as described in 1 and 3 above. Due to the large volume of treatment delivered, participants in this group may undergo a seventh visit with the physiotherapist to ensure adequate fit and comfort of the orthoses and understanding and progression of the physiotherapy exercise program.

All participants will receive an educational package at the commencement of the study, providing general information on PFP and advice on activity. In general terms, the advice on activity will entail an encouragement to participants to continue to exercise and participate in activities that do not provoke their knee pain, and to avoid aggravating activities particularly if the provoked pain persists longer than several minutes after cessation of the activity.

### Outcome measures

The blinded assessor will repeat the outcome measures following completion of the 6 visits with the physiotherapist at 6 weeks, and then at 12 and 52 weeks to assess the long term outcome of the intervention.

Outcome will be assessed at each time point using measures that have been previously demonstrated as being acceptably reliable and valid indictors of change [11-16] and used in previous high-quality RCTs of PFP [10,17,18]. The primary outcome measures will be as follows.

#### 1. Usual and worst pain Visual Analogue Scale

A 100 mm horizontal line with the descriptors 'no pain' at the 0 mm mark and 'worst pain imaginable' anchoring the 100 mm end will be used as a visual analogue scale. Participants will be required to place a vertical mark that represents their pain level on the horizontal line. They will do this twice, once to indicate their usual level of pain in the preceding week and the other to represent their worst pain experienced over the same time period. This will give two perceived pain severity ratings in millimetres [19]. The pain VAS has well-established reliability and validity in individuals with AKP [11,14,15,19].

#### 2. Anterior Knee Pain Scale

This scale consists of 13 items with discrete categories related to limp, weight bearing, walking, stairs, squatting, running, jumping, prolonged sitting with flexed knees, pain, swelling, painful patellar movements, thigh muscle atrophy, and flexion deficiency. One response to each item that best describes the participant's knee pain is selected and scored on a weighted basis, with the highest representing normal and asymptomatic [20]. The 13 individual items are then summed to provide a final score where 0 represents maximal disability and 100 represents no disability. This scale has been shown to have high test-retest reliability [15,16], moderate responsiveness to clinical change [16], and a demonstrated ability to discriminate between individuals with and without AKP [20].

#### 3. Functional Index Questionnaire

The Functional Index Questionnaire comprises eight questions pertaining to whether activities that are commonly problematic in PFP can be performed with or without difficulty [14,19]. The activities, including prolonged sitting, squatting and stair climbing, are each rated on a three point scale, where 0 = unable to do, 1 = can do with problem, and 2 = no problem. The sum of the scores for each of the eight activities provides an overall score, with 0 indicating maximal disability and 16 representing no disability. Studies have shown the FIQ to have fair to substantial test-retest reliability [11,14,15,19], and to be a valid measure for detection of clinical change [14].

#### 4. Patient Perceived Treatment Effect Score and Perceived Global Effect Visual Analogue Scale

Participants' perceived level of recovery will be rated on two scales at the 6, 12 and 52 week follow-up visits. Perceived Treatment Effect will be measured using a 5 point Likert scale with the following categories: (1) marked worsening, (2) moderate worsening, (3) same, (4) moderate improvement, and (5) marked improvement. For the purpose of analysis, this scale will then be dichotomised according to success, where 'success' is defined as marked or moderate improvement on this scale [10]. We will also conduct a sensitivity analysis on this dichotomisation. Perceived Global Effect Visual Analogue Scale will be piloted. Participants will rate their recovery by placing a vertical mark on a 200 mm VAS with 'same' in the middle to represent no change (0 mm), 'much worse' at the far left end (-100 mm) and 'completely better' as the right hand anchor (+100 mm). A positive score will represent improvement, while a negative score will indicate worsening of the condition.

Secondary outcome measures that will be taken include the following:

#### 1. Lower Extremity Functional Scale

The Lower Extremity Functional Scale is used to evaluate any difficulty experienced during daily activities as a result of the specific lower limb condition, such as PFP, and has been shown to be reliable, valid and sensitive to change in AKP participants [16] and those with general lower limb conditions [12]. Twenty items are rated individually on a 5 point scale indicating the degree of difficulty associated with performing that activity on the current day, ranging from 0 (extreme difficulty or unable to perform the activity) to 4 (no difficulty). Scores from each activity are summed to give an overall indication of functional difficulty, where 0 indicates maximal difficulty and 80 indicates no difficulty.

#### 2. Patient-Specific Functional Scale

The Patient Specific Functional Scale assesses individual disabilities in a short, efficient format, and was designed to complement generic or condition-specific measures [13]. It has been shown to have excellent test-retest reliability and sensitivity, and good validity in individuals with knee dysfunction [13]. Participants are asked to nominate up to 5 functional activities that they are experiencing difficulty with. The current level of difficulty associated with each activity due to the specified condition is then rated on an 11-point scale, where 0 is "unable to perform the activity" and 10 is "able to perform activity at same level as before injury or problem", and the average score across all activities is calculated.

#### 3. McGill Pain Questionnaire

The most commonly used multidimensional pain measure, the McGill Pain Questionnaire provides a description of the participant's pain experience, and has been utilised as a primary outcome measure in previous knee pain studies [21,22]. The first component involves descriptors of pain categorised as sensory, affective, evaluative and miscellaneous. Twenty groups of words are presented, with the participant required to select one word from each group that best describes their pain. The word chosen is scored by its rank order within the group, with the first word scoring 1, the second word scoring 2, and so forth. If no words are applicable a score of zero is recorded for that group. The Pain Rating Index is the sum of the scores across the 20 groups (range 0–78). The Number of Words Chosen is the number of groups for which a word was selected (out of 20). The second component is the Present Pain Index, a six point scale of severity ranging from no pain (0) to excruciating (5). For the final component, the participant selects one of three groups of words that best describes the pattern of their knee pain, or how pain changes with time, and leaves the question blank if there is no pain.

#### 4. Medical Outcomes Study 36-Item Short-Form Health Survey

This questionnaire is a widely-used generic measure of health-related quality of life [23], and has been previously used in PFP populations [10]. Thirty-six items are used to calculate eight multi-item scores: physical functioning, role limitations due to physical health problems, bodily pain, general health, vitality (energy/fatigue), social functioning, role limitations due to emotional problems, and mental health.

#### 5. Hospital Anxiety and Depression Scale

This 14-item scale will be used to investigate whether there is an association between PFP and emotional state, and has been found to be a reliable instrument for detection of anxiety and depression in an outpatient setting and a valid indicator of severity [24], and has been used in a previous study of physiotherapy in PFP [17]. Participants are required to select the best of 4 responses to questions pertaining to either anxiety or depression (7 questions each), which are scored from 0 to 3. The scores for the anxiety and depression questions are summed separately to give total scores for each component, where 0–7 represents no anxiety or depression, 8–10 is borderline, and 11–21 indicates the presence of an anxious or depressive state.

#### 6. Physical Activity Level in the Previous Week

Participants' physical activity levels will be quantified using a physical activity questionnaire by which occupational, household and leisure activities of varying intensities can be accurately and reliably measured [25]. This questionnaire involves calculation of the time spent in each of these activity types at moderate, hard and very hard intensities over the preceding 7 days. The total time in hours for each intensity of activity is then multiplied by the metabolic equivalents of the activities (METs, kcal/kg/hour), and summed to give an overall caloric output for the week, and divided by seven to give an average daily output. This can then be standardised to body weight to provide an index that can be compared between and within individuals across time [25]. This questionnaire has been shown to have moderate to high reliability on test-retest, especially with moderate and vigorous intensities of activity [25], and has been used previously in a PFP population [10].

#### 7. Step up, step down and squat tests

Three activities that typically provoke PFP will be used as a physical measure of function [10]. Step up and step down tests will be performed on a 25 cm step in a stepping order that continually loads the study knee, while squats will be of full excursion to the point where the fingers touch the floor. A metronome set at 96 beats per minute will be used to standardise the rate of testing. The repetition number on which the first onset of pain occurs, or the first increase in pain where a constant background ache is present, will be recorded, up to a maximum of 25 repetitions.

#### 8. Pressure Pain Threshold (PPT)

Pressure algometry, which has demonstrated reliability [26], will be used to measure PPT at four sites at the knee: (1) the proximal third of the medial border of the tibia; (2) the midpoint of the patella; (3) the distal portion of the rectus femoris muscle, 5 cm superior to the proximal border of the patella; (4) the most palpably tender point around the knee. A digital pressure algometer (Somedic AB, Farsta, Sweden) will measure the pressure applied at a rate of 40 kPa/s to the test site by a rubber-tipped probe (area 1 cm^2^) positioned perpendicular to the skin. The participant will activate a button at the precise moment that the pressure sensation changes to one of pressure and pain, which will signal cessation of pressure application and freeze the pressure reading onscreen for manual recording. Three measures will be taken at each site and the average calculated to represent the final value.

A further questionnaire regarding any adverse reactions to the intervention and whether any other treatment was sought will be completed at 12 months.

All participants will be required to maintain a daily diary over the 12 month period, recording activity levels, problems with the intervention, analgesia requirements and compliance with the home exercise program. Visual analogue scales for usual and worst pain over a week period will also be recorded.

### Sample size considerations

Sample size calculations for this study are based on the 100 mm visual analogue scale for usual pain over the past week. The mean difference between baseline and final score for each group will be compared to measure the effectiveness of each intervention. In order to detect a minimal clinically important difference of 15 mm [15,27], assuming a standard deviation of 20 mm [10], a power of 0.80 and alpha level of 0.01, a sample size of 40 participants per group will be required. To account for dropouts, particularly considering the long duration of the study [28], a group size of 44 will be recruited, with a total sample size of 176.

### Planned statistical analysis

Statistical analysis will be conducted on a blinded, intention to treat basis, with all participants who were initially randomised into the study included where data is available for each measurement time. SPSS software (Version 15.0) will be used for statistical procedures. The four groups will be examined for baseline comparability with respect to demographic data such as age, gender, body mass index and duration of knee pain, as well as baseline values of outcome measures. Continuous outcome measures taken at 6, 12 and 52 weeks will be analysed using univariate analysis of variance, where the baseline value of the outcome measure will be used as a covariate and group allocation as a fixed factor. Continuous data will also be expressed as area under the curve in order to compare the overall effectiveness of each intervention over the entire 12 month period. Participant demographics will also be included in the models as covariates to assess their impact on outcome. The dichotomous measure of success will be analysed using relative risk, risk reduction and numbers needed to treat in order to facilitate clinical interpretation of findings and future guidelines. Cost effectiveness analysis will evaluate the effectiveness of the intervention measured using Perceived Treatment Effect against resource usage (e.g. physiotherapist fees, equipment, medications, etc.), through calculation of the marginal cost effectiveness of treatment in terms of dollars per 1-point improvement in outcome. To accommodate for the possibility of inflated Type I error rate resulting from multiple comparisons, significance will be set at 0.01 (i.e., 99% confidence intervals).

## Discussion

In order to determine the clinical efficacy and cost-effectiveness of foot orthoses in the management of PFP, a pragmatic randomised clinical trial is to be conducted. Based on the Delphi List of criteria for quality assessment of RCTs [29], particular methodological factors have been incorporated into the study design to minimise bias and optimise the rigor of the RCT. Firstly, participants will be randomly allocated to intervention groups via concealed allocation. Poor allocation concealment has been shown to be associated with bias in RCTs [30]. Secondly, the investigator responsible for assessment of outcome at each time point will remain blind to participants' group allocation. Although it could be argued that the use of self-reported primary outcome measures may itself reduce bias associated with an unblinded assessor, blinding of outcome assessors is still an important component due to the potential for transfer of attitudes regarding an intervention from the assessor to the participant [31]. In this study, blinding of participants and the physiotherapists administering treatment is not possible due to the nature of the interventions used. However, as it has been recommended that greater credence should be placed in results where at least the investigators have been blinded to group allocation [31], the single-blind nature of this study is not deemed to be a methodological flaw. Thirdly, the investigators responsible for statistical analysis will be blind to the treatment group allocation, thereby minimising the likelihood of bias associated with their anticipated outcomes. Fourthly, the data analysis will proceed on an intention-to-treat basis, which amongst other things, maintains randomisation, conservatively manages inflation of type I error rate and imitates real life in which it is somewhat likely that not all patients will receive the ideal or intended treatment that has been prescribed.

As recommended by the CONSORT group [6], the RCT design has endeavoured to utilise outcome measures that have established reliability and validity and, where possible, have been used previously in PFP participants. This not only enhances the quality of the measurement and outcomes, but also facilitates direct comparisons with other studies that have investigated interventions for PFP and possible meta-analyses in the future. Furthermore, the selection of clinically applicable primary outcome measures that are easily administered in a clinical setting improves the clinical relevance of study findings.

The foot orthoses chosen for use in the RCT are a range of prefabricated devices that are widely used in Australian clinical practice. These orthoses were chosen over a custom moulded device on the basis of clinical time restraints, the higher direct cost of custom moulded orthoses, and findings from a recent meta-analysis of similar benefits for both types of devices in the management of lower limb overuse conditions [32]. In fitting the orthoses, we will adopt an approach based on a key synoptic paper by Nigg et al [33]. On the basis of evidence that does not support the traditional concept that inserts and orthoses are used to align the skeleton, they proposed a new concept for the function and fitting of shoes and shoe inserts. This new concept includes the following: (a) the skeleton has a preferred path for a given movement task; (b) if an intervention (shoe and/or insert) supports this preferred path, muscle activity can be reduced; (c) an optimal shoe and/or insert feels comfortable because it reduces muscle activity and the resulting fatigue; and (d) performance should increase with an optimal shoe and/or insert since muscle activity is minimised and thus energy expenditure is reduced. Therefore, the participants' perception of comfort will be used as the key guide for the selection and fitting of the appropriate orthoses. Although the orthoses are prefabricated, they do allow for a degree of modification in order to achieve best comfort fit. Once comfort has been achieved, using the steps outlined in Figure 2, if required, the device will be further modified to improve pain-free performance of a pain provocative functional task whilst still being comfortable. This is based on the proposition that the orthoses should minimise the patient's pain during function [34].

A particular feature of this study is the inclusion of flat inserts as an experimental control. This assumes that the shaped contoured form of the rear and mid-section of a moulded orthosis is its active constituent in controlling foot motion, especially excessive pronation. It is the control of motion that has traditionally been viewed by practitioners, and indeed intuitively by patients, as a major function of orthoses [35-37]. To the extent that the contoured form of an orthosis is conceptualised to be its active constituent, the control flat insert may then be regarded as a placebo. However, it has not been conclusively proven that orthoses do control motion, with some studies indicating no systematic effect on motion [38,39] and others to the contrary [40-42]. An alternative view is that foot orthoses simply serve as space fillers, with their shaped form facilitating full plantar contact [43], which is regarded by some to be clinically beneficial [44,45]. We contend that the inclusion of a flat insert as an experimental control condition is especially important due to the inconsistencies in findings and viewpoints surrounding foot orthoses.

In summary, the RCT to be conducted will utilise high-quality methodologies in accordance with CONSORT guidelines. It is anticipated that findings from this study will contribute to the limited knowledge base regarding the clinical efficacy of foot orthoses in the management of PFP, and provide clinicians with high-quality evidence upon which to base clinical decision making.

## Competing interests

In the past five years BV has received presenter fees from Vasyli International for the purpose of presenting seminars in the prescription and fitting of orthoses. The involvement in this project by Vasyli International is limited to the donation of the orthoses and inserts. Although BV conceptualised and has obtained funding from the NHMRC for the project and will act as overall co-ordinator of the project, he will be blinded to group allocation and he will not conduct the statistical analyses. As overall co-ordinator he will deal with all adverse events, in which case he will be unblinded for that individual so as to best manage and report the adverse event.

All other authors declare that they have no competing interests.

## Authors' contributions

NC and BV were responsible for writing this manuscript. BV is the sole chief investigator on the NHMRC grant #301037, which he conceived and wrote. TM, KC, EB and RD assisted in the study design. All authors have reviewed and approved this final manuscript.

## Pre-publication history

The pre-publication history for this paper can be accessed here:


